# A Novel Nanohybrid Nanofibrous Adsorbent for Water Purification from Dye Pollutants

**DOI:** 10.3390/ma9100848

**Published:** 2016-10-19

**Authors:** Shahin Homaeigohar, Ahnaf Usman Zillohu, Ramzy Abdelaziz, Mehdi Keshavarz Hedayati, Mady Elbahri

**Affiliations:** 1 Nanochemistry and Nanoengineering, Department of Chemistry and Materials Science and Engineering, School of Chemical Technology, Aalto University, Kemistintie 1, 00076 Aalto, Finland; ramzy.abdelaziz@aalto.fi; 2 Nanochemistry and Nanoengineering, Institute for Materials Science, Faculty of Engineering, University of Kiel, Kaiserstrasse 2, 24143 Kiel, Germany; ahnafuz@yahoo.com; 3 Nanochemistry and Nanoengineering, Helmholtz-Zentrum Geesthacht, Institute of Polymer Research, Max-Planck-Str. 1, 21502 Geesthacht, Germany; 4 Department of Micro- and Nanotechnology, Technical University of Denmark, Kongens Lyngby DK-2800, Denmark; mehhe@nanotech.dtu.dk

**Keywords:** nanofiber, nanocomposite, dye removal, water filtration, vanadium pentoxide (V_2_O_5_)

## Abstract

In this study, we devised a novel nanofibrous adsorbent made of polyethersulfone (PES) for removal of methylene blue (MB) dye pollutant from water. The polymer shows a low isoelectric point thus at elevated pHs and, being nanofibrous, can offer a huge highly hydroxylated surface area for adsorption of cationic MB molecules. As an extra challenge, to augment the adsorbent’s properties in terms of adsorption capacity in neutral and acidic conditions and thermal stability, vanadium pentoxide (V_2_O_5_) nanoparticles were added to the nanofibers. Adsorption data were analyzed according to the Freundlich adsorption model. The thermodynamic parameters verified that only at basic pH is the adsorption spontaneous and in general the process is entropy-driven and endothermic. The kinetics of the adsorption process was evaluated by the pseudo-first- and pseudo-second-order models. The latter model exhibited the highest correlation with data. In sum, the adsorbent showed a promising potential for dye removal from industrial dyeing wastewater systems, especially when envisaging their alkaline and hot conditions.

## 1. Introduction

The rapid increase of human population along with an exponential growth of industrialization have led to the increase of water demand over water supply globally [1,2]. This situation of water scarcity gives rise to a global challenge. One main reason for such a crisis is undoubtedly water pollution. Industries such as textiles, leather, paper, cosmetics, plastics, food, etc. are engaged with a dyeing process based on organic dyes and water. Disposal of dyes, which are in fact highly colored organic compounds and low biodegradable, into the water resources is a major cause of water pollution [1,3,4]. It is estimated that around 1%–20% of the total world production of dyes are lost during the dyeing process and released in the textile effluents [1,5]. Such toxic wastes adversely affect the metabolism of living organisms including human, environmental and natural processes like eutrophication [1,5].

To prevent the discharge of dyes into water resources, they have to be removed from wastewaters. For this purpose, coagulation, flocculation, biodegradation, adsorption, ion-exchange and advanced oxidation are the most commonly used techniques [3,4]. Among them, adsorption is accounted as the most effective and economical one [3,4,6].

Adsorbents such as fly ash, coal, peat, sawdust, lignite and wood have drawn considerable attention, mainly because of their availability and low cost [4]. Also, very recently, with a theoretically specified surface area as large as 2620 m^2^/g, graphene-based materials including graphene oxide [7] and reduced graphene oxide [8] have been studied for adsorption of cationic and anionic dyes through electrostatic and π-π interactions. Polymers are another class of adsorbents which possess distinct advantages when compared to classical adsorbents such as activated carbon and clay. For instance, they show favorable physicochemical stability, high selectivity and structural diversity, eco-friendliness and regeneration abilities [9,10,11]. Accordingly, they have found numerous applications in removal of cationic/anionic dyes [9,12,13,14].

Here, we aim to benefit from polyethersulfone (PES, a well-known polymeric membrane material) in an adsorption-based separation. In our study, PES is selected due to its high thermal and chemical resistance, as well as its appropriate mechanical properties [15]. Moreover, PES has an isoelectric point of 2.4–3.1 [16,17]. Therefore in basic conditions, hydroxyl groups emerge on its surface and interact with cationic dyes such as methylene blue (MB). Recalling the alkaline and hot conditions of wastewater streams of industrial dyeing processes, PES, due to its high thermal stability as well as hydroxylated surface at alkaline conditions, seems a very promising adsorbent material for cationic dyes.

To maximize the adsorptive property of PES, here we nanoengineer it physically and chemically. Physically, nanoengineering of PES as nanofibers brings about an extraordinary surface area. Hence, hydroxylation of the surface will be at its maximum level and a superior adsorption potential and dye removal capability is expected. While PES as a film has already been suggested for adsorption-based removal of dye pollutants [18,19,20], here, for the first time, the adsorptive potential of PES nanofibers will be precisely investigated. Moreover, chemically, to broaden the application range of the PES nanofibrous adsorbent to neutral and acidic conditions and higher operational temperatures, a nanoceramic with a lower isoelectric point is added to the nanofibers. Vanadium pentoxide (V_2_O_5_) is a functional ceramic currently stimulating immense research interest for employment in optical switching devices, catalysis, solar cell, sensors, etc. [21,22,23,24,25,26]. Considering a very low isoelectric point of 1–1.5 for V_2_O_5_ [27], in an aqueous medium, even at low pHs, its surface is hydroxylated, thus negatively charged [28], and can optimally catch cationic dyes. Therefore, for the first time, we report a polymeric nanofibrous adsorbent based on PES that is augmented in terms of dye adsorption ability as well as structural properties by inclusion of V_2_O_5_ nanoparticles.

## 2. Results and Discussion

### 2.1. Physicochemical Characteristics of the Nanofibrous Adsorbents

**Figure 1:**
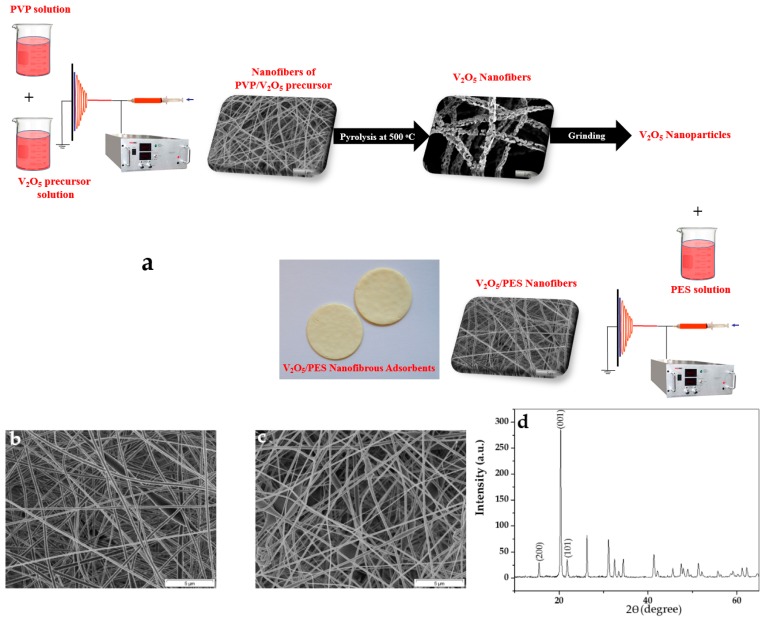
(**a**) Schematic of preparation procedure of the vanadium pentoxide/polyethersulfone (V_2_O_5_/PES) nanofibrous adsorbents; Morphology of the nanofibers of: (**b**) PES and (**c**) 5 wt % V_2_O_5_/PES; and (**d**) X-ray diffraction (XRD) pattern of the V_2_O_5_ nanoparticles after calcination at 500 °C. PVP: polyvinylpyrrolidone.

The V_2_O_5_ nanoparticles were prepared through a sol-gel process. In this regard, the V_2_O_5_ precursor was incorporated into a sacrificial nanofibrous matrix of polyvinylpyrrolidone (PVP), as shown in Figure 1a. Subsequently, calcination at the high temperature of 500 °C discarded the polymeric matrix and crystallized the nanofiller [29]. A combination of electrospinning and sol-gel can give rise to the creation of very fine and homogenously dispersed nanoparticles assembled as nanofibers [30]. In addition, the crystallized nanoparticles possess a higher surface energy and thereby an improved adsorption tendency [31]. The resulting mat was finally grinded to convert the nanofibers to distinct nanoparticles. As shown in Figure 1a, the V_2_O_5_/PES nanofibrous mats were produced by electrospinning of a PES solution (in DMAc) containing V_2_O_5_ nanoparticles (1, 3 and 5 wt %).

The morphology of the PES electrospun nanofibers in terms of bead formation, surface roughness and diameter distribution could affect the available surface area for adsorption. As shown in Figure 1b, the surface of the nanofibers is smooth, but numerous beads can be seen across the mat. This situation is also the case when the V_2_O_5_ nanoparticles are incorporated into the PES nanofibers (Figure 1c). Visually, the fiber diameter does not vary significantly between the compositions. At 0 wt % V_2_O_5_ (i.e., PES), the nanofibers’ diameter is 260 ± 110 nm, while at the highest filler concentration (i.e., 5 wt %) it decreases to 200 ± 56 nm. This finding indicates minor changes, i.e., increase of viscosity and viscoelastic property of the solution electrospun by addition of V_2_O_5_ [32,33,34,35].

The X-ray diffraction (XRD) results, as shown in Figure 1d, confirm that the V_2_O_5_ nanoparticles after calcination at 500 °C acquire an orthorhombic crystalline structure. The diffraction peaks appeared at 2θ = 16°, 20° and 22° for the V_2_O_5_ nanoparticles, are attributed to (200), (001) and (101) crystallographic planes of V_2_O_5_ crystallites [36].

**Figure 2:**
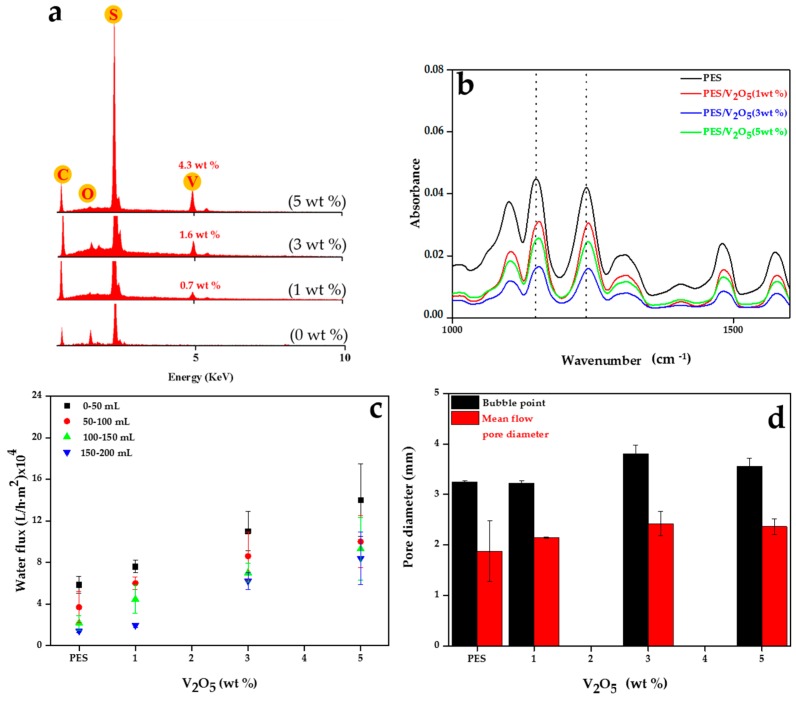
Chemical surface analysis of the PES electrospun nanofibrous adsorbents (ENAs) containing 0–5 wt % V_2_O_5_ nanoparticles through (**a**) energy dispersive X-ray spectroscopy (EDX) analysis and (**b**) attenuated total reflection Fourier transform infrared (ATR-FTIR); and different membrane characteristics of the PES ENAs including (**c**) water flux and (**d**) pore size.

The practical amount of the V_2_O_5_ nanoparticles incorporated into PES nanofibers was verified by energy dispersive X-ray spectroscopy (EDX) analysis. As seen in Figure 2a, other than 3 wt %, the practical value of the nanofillers is in harmony with the theoretical values. This implies that the nanoparticles could be homogenously distributed across and onto the nanofibrous mats. During electrospinning, the viscoelastic jets can possess significant initial longitudinal viscoelastic stresses generated in the preceding flow domain (the transition zone between the Taylor cone and the thin jet zone) [37]. Such stresses can disrupt the agglomerates of the nanoparticles and make a uniform dispersion of very fine nanoparticles.

Chemical surface analysis via attenuated total reflection Fourier transform infrared (ATR-FTIR) could imply the presence of and probable interaction between the ceramic nanofiller and the polymeric matrix. The ATR-FTIR of the PES electrospun nanofibrous adsorbents (ENAs) are shown in Figure 2b. The absorption peaks at 1296 and 1146 cm^−1^ are attributed to the asymmetrical and symmetrical vibrations of the sulfone group, respectively. The absorption peak at 1234 cm^−1^ is attributed to the stretching vibration of ether C-O-C bond in the PES polymer [38]. A comparison between ATR-FTIR spectra of the neat and composite ENAs reveal that the position of the peak at 1146 cm^−1^ for the neat PES ENA shifts to 1153 cm^−1^ for the V_2_O_5_/PES ENAs. Another shift is seen for the peak at 1234 cm^−1^ for the neat PES ENA, which is shifted to 1243 cm^−1^ for the V_2_O_5_/PES ENAs. Such shifts primarily indicate the presence of the V_2_O_5_ nanoparticles on/near to the surface of the nanofibers. These shifts are due to the interaction of the V_2_O_5_ nanoparticles with the PES matrix through a hydrogen bonding between the ceramic surface’s OH groups and ether C-O-C bond (or sulfone SO_2_ group) of PES [30,39,40,41]. Such a bonding could lead to a more optimal thermal and mechanical stability for the nanocomposite nanofibers, to be proved subsequently via thermogravimetric analysis (TGA) and compaction magnitude during a water permeability test [30].

Since, wastewater streams to be purified are mostly in a hot condition, the adsorbent material must be sufficiently thermally resistant. The chemical affinity of V_2_O_5_ nanoparticles towards the PES molecules, as verified by ATR-FTIR, could enhance thermal properties of the adsorbent. This hypothesis was probed by TGA. As shown in Table 1, such thermal characterization implies a meaningful increment in the thermal decomposition temperature (*T*_d_) for the nanocomposite ENAs versus the neat one. As mentioned earlier, the reason is most probably the interactions between the V_2_O_5_ nanoparticles and the polymer [42,43,44]. The hydrogen bond between the V_2_O_5_ nanoparticles and PES increases the rigidity of the polymer chain and thereby the energy of breaking it down [42]. On the other hand, some of the heat is absorbed by the V_2_O_5_ phase during heating-up, delaying the decomposition of PES and raising the decomposition temperature [45]. In this regard, the higher residual mass of the nanocomposite ENAs is a supportive finding. The same behavior was seen in our previous study [30]. Among the nanocomposite nanofibers, *T*_d_ has a descending trend with mass fraction of the filler. The reason could be a slight agglomeration of the nanoparticles, especially at the highest amount of the nanofiller, and thereby less uniform dispersion of them across the mat.

### 2.2. Structural Characteristics of the Nanofibrous Adsorbents

The PES nanofibrous adsorbent was designed to encompass the maximum possible amount of water. Such ultrahigh surface area can facilitate a reaction of the adsorbent material and the pollutant. The water permeability of the structure represents the available surface area and porosity of the adsorbent. As shown in Figure 2c, the PES ENA demonstrates a high water permeability of 2× 10^4^–6 × 10^4^ L/h·m^2^, which is quite larger than that of commercial microfiltration membranes [46]. This means a hydrodynamic adsorption-based separation could be done with a low energy consumption. As seen in Figure 2c, the permeability is enhanced via the nanocomposite strategy. The reasons for this behavior could be attributed to optimized physicochemical characteristics of the membranes and/or their porosity.

Of the physicochemical properties, mechanical stability and hydrophilicity are the most influential ones on water permeability. As proved through the water contact angle measurements, hydrophillicity of the ENAs does not vary by incorporation of the nanoparticles. The water contact angle remains in the range of 135°–140°. This effect can imply particles are partly buried under the surface and the rest are exposed on the surface. The exposed particles are not enough to induce a notable hydrophilicity effect. While incorporation of the nanoparticles does not confer a superior wettability to the ENAs, it can promote mechanical stability and lower the compaction tendency, thus optimizing water permeability [32]. The descending trend of water flux from 50 to 200 mL for each sample is attributed to compaction of the ENAs during the water flux measurement. The relevant magnitude is less notable for the nanocomposite ENAs, i.e., they are mechanically more resistant against disintegration induced by water flow stresses.

Based on porosity characteristics, the permeance behavior of the ENAs can be described according to Hagen–Poisseuille’s equation (Equation (1)) [47,48]:

(1) $$J=\frac{\varepsilon r^{2}}{8\mu \tau }\frac{\Delta P}{\Delta x}$$

where *J* is the water flux (m^3^/s), ε the porosity, *r* the pore radius (m), τ the tortuosity, Δ*P* the pressure difference across the membrane (Pa) (1 Pa = 10^−5^ bar), µ the dynamic viscosity (Pa·s) and Δ*x* the membrane thickness (m). Among the involved parameters in this equation, only porosity and pore size could be variable and directly influential on the water flux of the membranes. As measured by us, the porosity for all the samples varies in the range of 45%–60%. In addition, as shown in Figure 2d, the difference in pore size of all the samples, whether bubble point—i.e., the largest pore size—or mean flow pore diameter—the mean flow pore diameter is such that 50% of flow is through pores larger than the mean flow pore diameter and 50% of flow is through pores smaller than the mean flow pore diameter [15]—is not that significant. Therefore, the higher water permeability of the nanocomposite ENAs could be solely attributed to their more optimal mechanical stability induced by the presence of the nanoparticles.

### 2.3. Dye Removal Capacity of The Nanofibrous Adsorbent

Dye removal capacity of the PES ENA was probed while monitoring the effect of environmental factors (pH and temperature). Such a capacity was compared with that of the nanocomposite ENAs, as well.

#### 2.3.1. Effect of pH on Dye Removal

**Figure 3:**
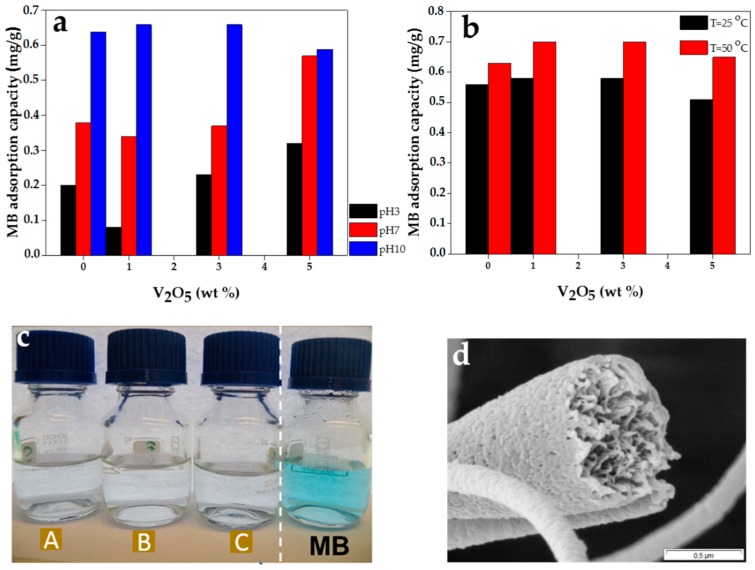
Methylene blue (MB) adsorption capacity of ENAs versus V_2_O_5_ content considering the effect of (**a**) pH and (**b**) temperature (at alkaline pH); (**c**) camera image of the purified aqueous solutions after adsorption process at hot and alkaline conditions versus the primary MB solution (A–C: treated by PES, 1 wt % V_2_O_5_/PES and 5 wt % V_2_O_5_/PES ENA, respectively); (**d**) porous internal and external structure of the nanocomposite nanofibers (5 wt %) facilitating the diffusion of MB solution upon temperature rise.

The plot of dye removal capacity of the PES ENA with respect to the pH of the medium is shown in Figure 3a. As deduced from the plots, while in basic pH the ENA adsorbs MB dye molecules significantly, in acidic as well as neutral pH the dye adsorption is low. Since the wastewaters discharged from dyeing processes are inherently alkaline [4,49], the PES ENA shows a desirable dye removal performance at this condition. As seen in Figure 3a, the addition of V_2_O_5_ nanoparticles does not make any big difference in adsorption capacity. Only when the nanofiller amount increases to 5 wt % the nanocomposite ENAs show a superior adsorption performance than the neat one does in acidic and neutral conditions.

The significant role of pH on adsorption performance of an adsorbent has been already stated in numerous studies [3,50]. For instance, Crini et al. [51] declare that the pH of the solution affects not only the surface charge and functional groups of the adsorbent, but also the degree of ionization of the dye in the solution and the solution chemistry.

The optimized adsorption in basic pH is described through the following mechanism:

MB molecules are dissociated in aqueous media as:

(2) C_16_H_18_N_3_SCl → C_16_H_18_N_3_S^+^ + Cl^−^

In basic condition, the surface hydroxyl group of PES as well as V_2_O_5_ is de-protonated as:

(3) PES/V_2_O_5_–OH + OH^−^ → PES/V_2_O_5_–O^−^ + H_2_O

Accordingly, through the electrostatic attraction between the negatively charged oxygen atom and the dye cation, the adsorption process occurs.

(4) PES/V_2_O_5_–O^−^ + C_16_H_18_N_3_S^+^ → PES/V_2_O_5_–O^−^–S^+^–N_3_H_18_C_16_

The higher degree of de-protonation for hydroxyl groups in basic condition will likely result in an enhanced chemisorption between the dye and PES/V_2_O_5_. Remembering an isoelectric point of 2.4–3.1 for PES [16,17], in basic condition hydroxyl groups emerge on the surface of PES and thereby interact with the cationic dye. However, in lower pHs of three and seven, a lesser density of surface hydroxyl groups decreases the electrostatic interaction between the dye and the nanofibers. A similar result has been reported for adsorption of MB onto perlite [50].

#### 2.3.2. Effect of Filler Concentration on Dye Removal

As seen in Figure 3a,b, in basic condition, the PES ENA adsorbs MB molecules as much as the V_2_O_5_/PES ENAs do. This finding can be clearly seen in Figure 3c, indicating a perfect absorption of MB from water by all the adsorbents regardless of the nanoparticle content. The reason could be a similar saturated state of the hydroxyl group on both the samples’ surfaces. The pH is high enough and far above isoelectric point of the components of the system. This situation induces a full hydroxylation of the surfaces. However, in acidic and neutral conditions, the composition of the ENAs matters. While for the samples containing 0–3 wt % V_2_O_5_, the adsorption capacity is almost equal, for the sample richest in terms of the V_2_O_5_ nanofiller, the adsorption capacity rises markedly. This implies that at lower pHs, the higher V_2_O_5_ concentration is, the higher the density of OH groups, i.e., reactive sites could emerge on the surface. Thus, a higher electrostatic interaction and adsorption capacity is acquired. In fact, owing to the much lower isoelectric point of V_2_O_5_, the surface of 5 wt % V_2_O_5_/PES nanofibers are in a wider pH range negatively charged than the other samples and thereby a higher adsorption occurs. However, the low adsorption capacity at acidic and neutral pHs even for the highest content of V_2_O_5_ could be attributed to much less hydroxylation of the ENAs compared to basic condition.

#### 2.3.3. Effect of Temperature on Dye Removal

As shown in Figure 3b, at a higher temperature, the PES ENA adsorbs more MB molecules. This behavior is the case for the nanocomposite system as well. This indicates that the adsorption process is endothermic. Such a behavior could be induced by a promoted mobility of the MB ions with increasing temperature and their interaction with the nanofibers [52]. Indeed, here, heating compensates for the lack of stirring and agitation of the solution during our experiment. Moreover, increasing temperature may lead to a swelling effect of the internal/external structure of the nanofibers (Figure 3d), facilitating diffusion of the dye solution into the surface pores. Also, from a chemistry point of view, upon rise of temperature, the chemisorption reaction between the surface hydroxyl groups of our adsorbent system and the cationic group in the MB molecules eases [50]. Similar results were reported for the adsorption of Congo red onto fly ash [52], Astrazone blue onto hardwood sawdust [53], and for the adsorption of MB onto unexpanded perlite [54].

### 2.4. Adsorption Thermodynamics

To comprehend the effect of temperature on the adsorption, the thermodynamic parameters such as standard Gibbs free energy Δ*G*^0^, standard enthalpy Δ*H*^0^, and standard entropy Δ*S*^0^ should be studied.

Δ*G*^0^ is determined from the following equation [4,52]:

(5) Δ*G*^0^ = −*RT lnK_c_*

where *K_c_* is the adsorption equilibrium constant and correlated to Δ*H*^0^ and Δ*S*^0^ of adsorption by the van’t Hoff equation:

(6) $$lnK_{c}=\frac{-\Delta H^{0}}{RT}+\frac{\Delta S^{0}}{R}$$

where *R* is the gas constant and *T* the temperature. The *K_c_* value is calculated from Equation (7):

(7) *K_c_* = *C*_Ae_/*C*_Se_

where *C*_Ae_ and *C*_Se_ are the equilibrium concentration (mg/L) of the dye ions on the adsorbent and in the solution, respectively. The van’t Hoff plots (*lnK_c_* vs. 1/*T* (kelvin)) for the adsorption of MB onto PES ENAs (not shown here) were used for calculation of Δ*H*^0^ and Δ*S*^0^. The slope is equal to −Δ*H*^0^*/R* and its intercept to Δ*S*^0^*/R*. All the thermodynamic parameters obtained are presented in Table 2. As shown in the table, the negative values of Δ*G*^0^ at basic pHs at both temperatures indicate the spontaneous nature of the adsorption process. The degree of spontaneity also increases with temperature. A similar behavior was observed in other researches [55,56].

In all conditions in terms of composition, pH and temperature, Δ*H*^0^ is positive, indicating an endothermic adsorption [55]. This fact has been reported by other researchers as well [55,57,58]. The positive values of Δ*S*^0^ in all the conditions suggest the increased randomness at the solid/solution interface during the adsorption due to redistribution of energy between MB and the PES nanofibers, i.e., the affinity of the adsorbent for MB [55,56,59]. A similar trend has been reported for the adsorption of Congo red onto activated carbon and also some reactive dyes onto aluminium hydroxide sludge adsorbents [55,57].

### 2.5. Adsorption Kinetics

**Figure 4:**
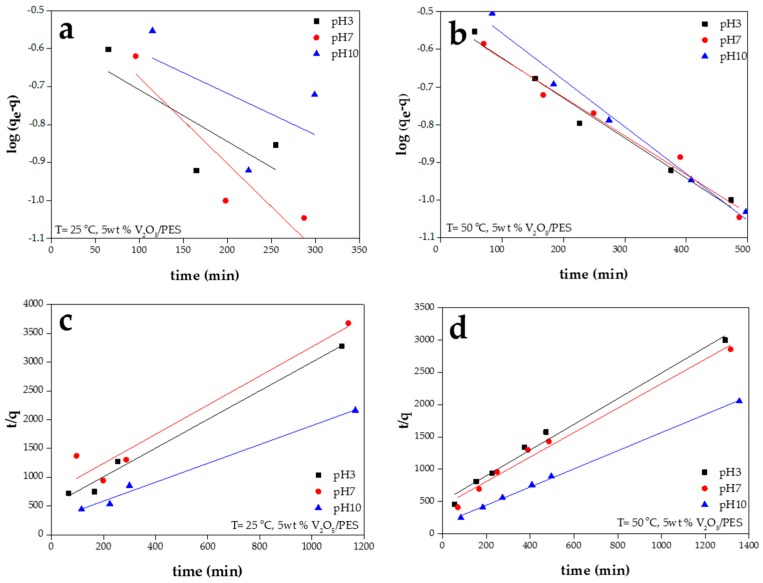
Plots of 1st (**a**,**b**) and 2nd order (**c**,**d**) kinetic models of MB adsorption onto the (5 wt %) V_2_O_5_/PES ENAs (plot **a** clearly implies lack of harmony of the adsorbent system at the low temperature of 25 °C with the Lagergen model).

The efficiency of adsorption and applicability of scale-up operation is determined by kinetic study of the adsorption process [3]. In this regard, pseudo-first-order and second-order kinetic models were used to gain a better understanding of the adsorption process. First, the kinetic data were fitted to the first-order kinetic model of Lagergen [57]:

(8) *log*(*q_e_* − *q*) = *logq_e_* − *k*_1_*t*/2.303

where *q_e_* and *q* are the amounts of dye adsorbed (mg/g) at equilibrium and at time *t* (min), respectively, and *K*_1_ is the rate constant of adsorption (1/min). Values of *K*_1_ were calculated from the plots of *log(q_e_* − *q)* vs. *t* (e.g., Figure 4a,b related to 5 wt % V_2_O_5_/PES that shows the most optimal adsorption capacity at all pH levels) for different samples. An *r*^2^ value approaching 1 as well as a good agreement between the experimental *q_e_* values with the ones calculated from the linear plots (Table 3) are indications of a proper harmony with the Lageregen model and first-order interaction of MB and the adsorbents. Accordingly, mostly at the high temperature of 50 °C and mostly at higher pH values, the adsorption of MB onto the V_2_O_5_/PES nanofibers is a first-order reaction [57]. For the other conditions in terms of pH and temperature, the kinetic data were further modeled with the pseudo second-order kinetic equation.

The second-order kinetic model is expressed as [57,60,61]:

(9) *t/q* = 1/(*k*_2_*q_e_*^2^) + *t*/*q_e_*

where *k*_2_ (min g/mg) is the rate constant of second-order adsorption. In the case of a linear plot of *t/q* versus *t*, the kinetic is of a second order. The second-order constants *k*_2_ and *q_e_* were calculated from the intercept and slope of the plots. At the lower temperature of 25 °C regardless of pH as well as at the high temperature of 50 °C at pH10 and in some cases pH7, the linear plots of *t/q* vs. *t* show a good agreement of experimental data with the second-order kinetic model for different samples (e.g., Figure 4c,d). The *r*^2^ values for the second-order kinetic model are mostly greater than 0.98 (Table 3). In addition, the calculated *q_e_* values comply very well with the experimental ones. Thus, the adsorption system at such conditions behaves as the second-order kinetic model. Accordingly, it is assumed that the rate limiting step may be chemisorption, involving valency forces through sharing or exchange of electrons between sorbent and sorbate [61]. A similar behavior is observed in the biosorption of dye Remazol Black B on biomass [62,63] and adsorption of Congo red on activated carbon prepared from coir pith [57].

### 2.6. Adsorption Isotherms

The equilibrium isotherm is of significant importance to understand the behavior of adsorption process and the affinity of dye molecules [3]. The analysis of equilibrium data for the adsorption of MB onto the PES ENAs was performed considering the Freundlich isotherm model [52,57]. Assuming that the adsorbent surface is heterogeneous, the Freundlich adsorption isotherm is expressed as [4,52]:

(10) $$\text{log}\;q=\text{log}\;K_{f}+\frac{1}{n}\text{log}\;C_{e}$$

(11) $$q=\frac{\left(C_{i}-C_{e}\right)v_{\text{sol}}}{m}\times 10^{-3}$$

where *q* is the amount of dye adsorbed (mg) per gram of the adsorbent, *C*_e_ and *C*_i_ are the equilibrium and initial concentrations (mg/L), *m* is the adsorbent mass used (g), *V*_sol_ is the solution volume (L) and *K_f_* and *n* are isotherm constants indicating the capacity and intensity of the adsorption, respectively.

The plots of log*q* versus log*C*_e_ at different pHs and two temperatures of 25 and 50 °C (not shown here) were used for calculation of *K_f_* (slope) and *n* (intercept) isotherm constants (as shown in Table 4). According to the values of the correlation coefficient (adjusted *r^2^*) of the plots (Table 4), unlike in basic condition (wherein the adsorbent surface is homogenous due to saturation of –OH groups), the plots at acidic and especially neutral pHs are in a good harmony with the Freundlich adsorption model. The dye adsorption capacity of the adsorbent is directly proportional to the *K_f_* values [57]. Increase of the value of *K_f_* with temperature indicates that the adsorption process is endothermic [55]. On the other hand, when 0 < (1/*n*) < 1, the adsorption is suitable [55] which was applicable only in basic condition.

## 3. Materials and Methods

### 3.1. Materials

PES (Ultrason E6020P; *M*_w_ = 58,000 and density of 1.37 g/cm^3^) was purchased from BASF (Ludwigshafen, Germany). The solvents of ethanol, *N*,*N*-dimethylacetamide (DMAc) and acetic acid were obtained from Merck (Darmstadt, Germany). To produce V_2_O_5_ nanoparticles via a sol-gel process, vanadium oxide precursor (vanadium tri-isopropoxide oxide) was supplied from Alfa Aesar GmbH & Co. KG (Karlsruhe, Germany). Polyvinylpyrrolidone (PVP) (*M*_w_ = 1,300,000) and MB were purchased from Sigma-Aldrich (St. Louis, MO, USA).

### 3.2. Preparation of the Nanofibrous Adsorbents

The PES nanofibrous adsorbents were produced by electrospinning. Briefly, prepared PES solution (20 wt %) in DMF was fed with a constant rate of 0.5 mL/h into a needle using a syringe pump (Harvard Apparatus, Holliston, MA, USA). By applying a 20 kV voltage (Heinzinger Electronic GmbH, Rosenheim, Germany) PES was electrospun on aluminum foil, then peeled off. The electrospinning conditions are tabulated in Table 5.

The nanocomposite nanofibrous adsorbents were made by inclusion of V_2_O_5_ nanoparticles into PES nanofibers. Beforehand, the V_2_O_5_ nanoparticles were prepared through a sol-gel process. In this regard, the V_2_O_5_ precursor was incorporated into a sacrificial nanofibrous matrix of PVP. Calcination at a high temperature discards the polymeric matrix and crystallizes the ceramic nanofiller [29].

The V_2_O_5_ precursor nanofibers were made by electrospinning of a mixture of vanadium tri-isopropoxide oxide solution (20 wt % in ethanol/acetic acid (1:1)) and PVP solution (10 wt % in ethanol) under the conditions tabulated in Table 5. The ratio of the first to second solutions was 2:1. Subsequently, the nanofibers were calcinated at 500 °C in air for 1 h to end up in a V_2_O_5_ nanofibrous mat. The resulting mat was finally grinded to convert the nanofibers to distinct nanoparticles.

The V_2_O_5_/PES nanofibrous mats were produced by electrospinning of a PES solution (in DMAc) containing V_2_O_5_ nanoparticles (1, 3 and 5 wt %) under the conditions tabulated in Table 5. To verify the practical amount of the nanoparticles, an EDX analysis was performed.

### 3.3. Characterization of the Physicochemical Properties

The morphology of the nanofibers in terms of bead formation, surface morphology and diameter distribution was probed by scanning electron microscopy (SEM) (LEO 1550VP Gemini from Carl ZEISS). The diameter of the nanofibers and V_2_O_5_ nanoparticles were assessed from SEM images using the Adobe Acrobat v.07 software.

To investigate presence and probable interaction between the nanoparticles and polymer matrix, chemical surface analysis of the nanofibers was performed by Fourier transform infra red spectrometry (FTIR). Attenuated total reflection Fourier transform infrared (ATR-FTIR) spectra were recorded using a Bruker Equinox 55 spectrometer. Additionally, to prove crystallization of the V_2_O_5_ nanoparticles by calcination at 500 °C, structural analyses of them were carried out at room temperature using an X-ray diffractometer (XRD3000TT, Agfa Gevaert (previously RICH. SEIFERT & Co GmbH), Mortsel, Belgium) with Cu- Kα radiation (λ = 0.1541 nm).

Thermal stability of the adsorbents, which could be determining in their performance in relevance to hot industrial wastewaters, was investigated by TGA. This characterization was carried out with a thermogravimetric analyzer of Netzsch 209 TG. TGA analysis was performed at 20–1000 °C with a heating rate of 10 °C/min under Argon. The decomposition temperature (*T*_d_) was defined as the temperature at 5% weight loss.

### 3.4. Characterization of the Structural Properties

The porosity of the PES ENAs, representing the available surface area for the adsorption process, was calculated according to Equation (12) [64]:

(12) $$\varepsilon =\left(1-\frac{\rho }{\rho_{0}}\right)\times 100\%$$

where ε is porosity, ρ_0_ and ρ are the average density of the materials used in electrospinning and apparent density of the electrospun mats, respectively. ρ_0_ can be calculated based on the following Equation (13):

(13) $$\frac{1}{\rho_{0}}=\frac{\varphi_{\text{PES}}}{\rho_{\text{PES}}}+\frac{\varphi_{V_{2}O_{5}}}{\rho_{V_{2}O_{5}}}$$

where $\rho_{\text{PES}}$ and $\rho_{V_{2}O_{5}}$ are 1.37 and 3.36 g/cm^3^, respectively. $\varphi_{\text{PES}}$ and $\rho_{\text{PES}}$ are mass fractions of the components.

The pore size of the ENAs was characterized through a liquid-gas displacement method so-called “bubble point” test [15].

Wettability of the ENAs was assessed through water contact angle measurement, using a contact angle analysis system (Kruess DSA 100, Hamburg, Germany). A 5 µL droplet was dispensed on the membrane and the resultant angle was measured.

To appraise the water permeability of the ENAs, a water flux measurement was performed. In this test, the circular membranes (*d* = 20 mm) were stamped out and placed over a poly(*p*-phenylene sulfide) (PPS) technical nonwoven support layer. The hybrid membranes were subsequently put in the membrane module of a custom-built set-up (shown in [30]) and the amount of the water passed through them under a 0.5 bar pressure after 50, 100, 150 and 200 s was measured. The water flux was calculated by Equation (14):

(14) $$J=\frac{Q}{A\cdot \Delta t}$$

where *J* is the water flux (L/h·m^2^), *Q* is the permeated volume of water (L), *A* is the effective area of the ENAs (m^2^) and Δ*t* is the sampling time (h).

### 3.5. Characterization of the Dye Removal Efficiency

Briefly, 30 mg of the ENAs was immersed in 30 mL aqueous solution of MB (1 mg/L) without stirring. The solution samples were collected after one overnight immersion to reach to an equilibrium state. The dye concentration was analyzed by monitoring their absorbance using UV–Vis spectrophotometer (UV-1800 Shimadzu, Japan). The experiments were carried out as a function of V_2_O_5_ content (0, 1, 3 and 5 wt %), pH (3, 7 and 10) and temperature (25 and 50 °C). The pH of the solution was adjusted by addition of proper amounts of acetic acid (to acidify) and ammonium hydroxide (to basify).

The dye removal percentage was calculated using the following Equation (15) [3]:

(15) $$\text{Removal}\;(\%)=\frac{C_{i}-C_{e}}{C_{i}}\times 100$$

where *C*_i_ and *C*_e_ are the initial and equilibrium dye concentrations (mg/L), respectively.

## 4. Conclusions

The dye removal potential of a new polymeric nanofibrous adsorbent was studied. The adsorbent showed an optimum capability in the removal of MB especially at hot and alkaline conditions. Temperature has an increasing effect on dye removal capacity of the nanofibrous adsorbent implying the endothermic nature of the adsorption process. In acidic and neutral conditions, the adsorption isotherm follows the Freundlich model, implying a non-homogenous adsorbent layer, while at alkaline condition the behavior of the adsorbent does not comply with this model, attributed to the formation of a homogenous adsorbent layer. Accordingly, regardless of the composition of the adsorbent (i.e., as pristine or nanocomposite) the highest adsorption capacity was found to be 85% in basic conditions and high temperature. These environmental parameters (i.e., basic and high temperature) are indeed realistic conditions for industrial wastewaters of dyeing processes. The results would be useful for the fabrication and designing of wastewater treatment plants for the removal of dye.

## Figures and Tables

**Table 1:** Thermal properties of the nanofibrous adsorbents produced in this study.

The Nanofiller Amount	Thermal Decomposition Temperature (°C)	Residual Mass (%)
0 wt %	466	1.7
1 wt %	493	3.5
3 wt %	486	4.3
5 wt %	478	7.4

**Table 2:** Thermodynamic parameters for the adsorption of methylene blue (MB) onto the polyethersulfone (PES) electrospun nanofibrous adsorbents (ENAs).

Temperature (°C)	*K*_c,25°C_	*K*_c,50°C_	Δ*G*^0^ (kJ/mol), 25 °C	Δ*G*^0^ (kJ/mol), 50 °C	Δ*H*^0^ (kJ/mol), 25 °C	Δ*H*^0^ (kJ/mol), 50 °C	Δ*S*^0^ (kJ/mol), 25 °C	Δ*S*^0^ (kJ/mol), 50 °C
PES (pH3)	0.267	0.31	3.27	3.14	0.005	0.005	0.005	0.005
PES (pH7)	0.153	0.32	5.04	3.14	0.025	0.025	0.06	0.06
PES (pH10)	1.72	3.43	−1.343	−3.30	0.023	0.023	0.08	0.08
1 wt % V_2_O_5_/PES (pH3)	0.1	0.29	5.7	3.32	0.03	0.03	0.1	0.1
1 wt % V_2_O_5_/PES (pH7)	0.1	0.3	5.7	3.32	0.03	0.03	0.1	0.1
1 wt % V_2_O_5_/PES (pH10)	1.81	4.38	−1.47	−3.96	0.03	0.03	0.1	0.1
3 wt % V_2_O_5_/PES (pH3)	0.33	0.475	2.74	2	0.01	0.01	0.03	0.03
3 wt % V_2_O_5_/PES (pH7)	0.135	0.184	5	4.54	0.01	0.01	0.02	0.02
3 wt % V_2_O_5_/PES (pH10)	1.705	3.1	−1.32	−3.04	0.02	0.02	0.07	0.07
5 wt % V_2_O_5_/PES (pH3)	0.607	0.87	1.23	0.373	0.01	0.01	0.03	0.03
5 wt % V_2_O_5_/PES (pH7)	0.525	1.02	1.6	−0.053	0.02	0.02	0.07	0.07
5 wt % V_2_O_5_/PES (pH10)	1.5	3.31	−1	−3.21	0.03	0.03	0.09	0.09

**Table 3:** Comparison of the first- and second-order adsorption kinetic models for the PES ENAs (exp and cal denote experimental and calculated amounts of dye adsorbed at equilibrium (*q_e_*), respectively).

Parameter	1st Order	Kinetic	Model		2nd Order	Kinetic	Model
	*q_e_* (exp) (mg/g)	*K*_1_ (1/min)	*q_e_* (cal) (mg/g)	*r*^2^	*K*_1_ (1/min)	*q_e_* (cal) (mg/g)	*r*^2^
PES, pH3, *T* = 25 °C	0.19	0.002	0.145	−0.38	0.014	0.24	0.93
PES, pH7, *T* = 25 °C	0.12	0.002	0.042	−0.395	0.1	0.127	0.99
PES, pH10, *T* = 25 °C	0.57	0	0.15	−1	0.02	0.61	0.98
PES, pH3, *T* = 50 °C	0.202	0.001	0.25	0.7	0.002	0.41	0.55
PES, pH7, *T* = 50 °C	0.206	0.001	0.134	0.9	0.01	0.29	0.91
PES, pH10, *T* = 50 °C	0.658	0.002	0.4	0.98	0.008	0.75	0.99
1 wt % V_2_O_5_/PES, pH3, *T* = 25 °C	0.08	0	0.046	−0.99	0.02	0.11	0.66
1 wt % V_2_O_5_/PES, pH7, *T* = 25 °C	0.08	0.009	0.09	0.56	0.1	0.08	0.98
1 wt %V_2_O_5_/PES, pH10, *T* = 25 °C	0.58	0.005	0.467	0.67	0.013	0.64	0.99
1 wt % V_2_O_5_/PES, pH3, *T* = 50 °C	0.2	0.002	0.275	0.93	0.002	0.4	0.55
1 wt % V_2_O_5_/PES, pH7, *T* = 50 °C	0.196	0.002	0.144	0.99	0.016	0.245	0.99
1 wt %V_2_O_5_/PES, pH10, *T* = 50 °C	0.692	0.003	0.436	0.99	0.01	0.763	0.99
3 wt % V_2_O_5_/PES, pH3, *T* = 25 °C	0.23	0.005	0.275	0.98	0.01	0.284	0.99
3 wt % V_2_O_5_/PES, pH7, *T* = 25 °C	0.11	0.008	0.206	0.95	0.027	0.14	0.87
3 wt %V_2_O_5_/PES, pH10, *T* = 25 °C	0.58	0.002	0.363	0.98	0.012	0.63	0.99
3 wt % V_2_O_5_/PES, pH3, *T* = 50 °C	0.29	0.005	0.34	0.97	0.013	0.35	0.99
3 wt % V_2_O_5_/PES, pH7, *T* = 50 °C	0.14	0.001	0.151	0.7	-	-	−0.43
3 wt %V_2_O_5_/PES, pH10, *T* = 50 °C	0.68	0.003	0.575	0.95	0.006	0.8	0.99
5 wt % V_2_O_5_/PES, pH3, *T* = 25 °C	0.34	0.002	0.267	0.182	0.012	0.4	0.98
5 wt % V_2_O_5_/PES, pH7, *T* = 25 °C	0.31	0.005	0.354	0.71	0.009	0.39	0.91
5 wt %V_2_O_5_/PES, pH10, *T* = 25 °C	0.54	0.002	0.316	−0.4	0.01	0.61	0.98
5 wt % V_2_O_5_/PES, pH3, *T* = 50 °C	0.4	0.002	0.301	0.98	0.008	0.5	0.98
5 wt % V_2_O_5_/PES, pH7, *T* = 50 °C	0.43	0.002	0.301	0.97	0.004	0.53	0.98
5 wt %V_2_O_5_/PES, pH10, *T* = 50 °C	0.65	0.002	0.371	0.98	0.01	0.71	0.99

**Table 4:** Freundlich isotherm constants for the adsorption of MB onto the V_2_O_5_/PES ENAs.

	*K_f_* (mg/g)	1/*n* (L/g)	*r*^2^
*T* = 25 °C, pH3	0.05	3.70	0.82
*T* = 50 °C, pH3	0.1	1.75	0.80
*T* = 25 °C, pH7	0.05	3.70	0.94
*T* = 50 °C, pH7	0.1	1.85	0.99
*T* = 25 °C, pH10	0.3	0.6	0.57
*T* = 50 °C, pH10	0.6	0.1	0.21

**Table 5:** Electrospinning conditions of the nanofibers produced in this study.

Electrospinning Conditions	PES	V_2_O_5_ Precursor/PVP	V_2_O_5_/PES
Voltage (kV)	20	17	30
Spinning distance (cm)	20	25	20
Collector	Al foil	Al foil	Al foil
Feed rate (mL/h)	0.5	0.5	3
Polymer concentration (wt %)	20	10	21
